# Hand posture affects brain-function measures associated with listening to speech

**DOI:** 10.1038/s41598-020-59909-0

**Published:** 2020-02-25

**Authors:** Koichi Tsunoda, Mihiro Takazawa, Sotaro Sekimoto, Kenji Itoh, Thomas Baer

**Affiliations:** 1 National Institute of Sensory Organs, National Hospital Organization Tokyo Medical Center, Department of Artificial Organs and Medical Creations, Tokyo, 152-8902 Japan; 2 University of Cambridge, Department of Experimental Psychology, Cambridge, CB2 3EB UK

**Keywords:** Neurophysiology, Brain

## Abstract

A major difficulty in studies of the brain, from the molecular to large-scale network level, is ensuring the accuracy and reliability of results, since repeatability has been a problem in studies utilizing functional magnetic resonance imaging (f-MRI) near-infrared spectroscopy (NIRS), and positron-emission tomography (PET). More generally, an effort to replicate psychological studies has shown that the original results were unambiguously reobtained only 39% of the time. It has been suggested that researchers must undertake studies to identify factors that reduce reliability and conduct more carefully controlled studies to improve reliability. In our previous work, we examined whether changes in hand/arm posture can have a confounding effect on task-related brain activity. Here we show a solution to enhance reproducibility in a NIRS study in a hearing task. The results showed that crossed posture can lead to different results than parallel posture with respect to asymmetric functional connectivity, especially during non-resting state. Even when the only task is listening to speech stimuli, participants should be asked to place their hands on a surface and feet on the floor and keep the same stable posture to increase reproducibility of results. To achieve accurate reliability and reproductively of results, stable hand posture through the experiment is important.

## Introduction

A major difficulty in studies of the brain, from the molecular to large-scale network level, is ensuring the accuracy and reliability of results, since repeatability has been a problem in studies utilizing functional magnetic resonance imaging (f-MRI), near-infrared spectroscopy (NIRS), and positron-emission tomography (PET)^1^. More generally, an effort to replicate psychological studies has shown that the original results were unambiguously reobtained only 39% of the time^2^. It has been suggested that researchers must undertake studies to identify factors that reduce reliability and conduct more carefully controlled studies to improve reliability^3^.

Uncontrolled posture may be a factor contributing to the failure to replicate. Previous reports have indicated that volunteers’ posture without attention could affect the outcomes of physiological studies^4,5^. Another report showed that crossing the hands over the midline impairs the ability to correctly judge the order of a pair of tactile stimuli when they are delivered in rapid succession to each hand. This impairment, termed the “crossed-hands deficit,” has been attributed to a mismatch between the somatotopic and body-centered frames of reference, onto which somatosensory stimuli are automatically mapped^6^. There were some reports that the position affects the result in physiological studies. Hand position and visual feedback^7^, difference of sensory processing across hand configurations^8^ and task-irrelevant sounds influence both temporal order and apparent-motion judgments about tactile stimuli applied to crossed and uncrossed hands^9^. In the clinical setting hand posture has been evaluated in a possible novel approach to the treatment of painful clinical conditions Crossing the arms over the midline was found to impair the multimodal processing of somatosensory stimuli and induce significant analgesia to noxious hand stimulation^10^. In our previous study, we examined whether changes in hand/arm posture can have a confounding effect on task-related brain activity^11^. We examined cerebral blood flow, which reflects neuronal activation, using NIRS in volunteers who were seated in front of a desk with their hands on the desk and feet firmly on the floor. At regular intervals, they were instructed to switch between normal (parallel arm) and crossed positions of the hands while otherwise maintaining a stable posture. Effects of posture (two different hand postures) were observed, indicating that postural adjustments could compromise the satisfactory replication of the results of brain studies^11^.

In the present study, we build on our previous result by adding a condition involving auditory perception. We monitored brain activity while participants heard either speech or silence and we analyzed the differences of brain activity during normal (parallel arm) and crossed positions of the hands, with otherwise stable posture. Results show that changes of hand posture cause subtle changes that can influence conclusions about laterality of brain activity during speech perception. Experimenters in similar research topics should require subjects to maintain stable posture with hands on the table and feet on the floor to improve the reliability and reproducibility of results.

## Result

### The general effect of hand posture on channel activity

Participants (17 in all) were asked to sit with hands on a table, either in a normal posture or with hands/arms crossed, and feet on the floor. Data were collected from 16 NIRS channel arrayed across the forehead and sampling the prefrontal cortex. They listened to silence or speech in alternating data-recording epochs.

**Figure 1:**
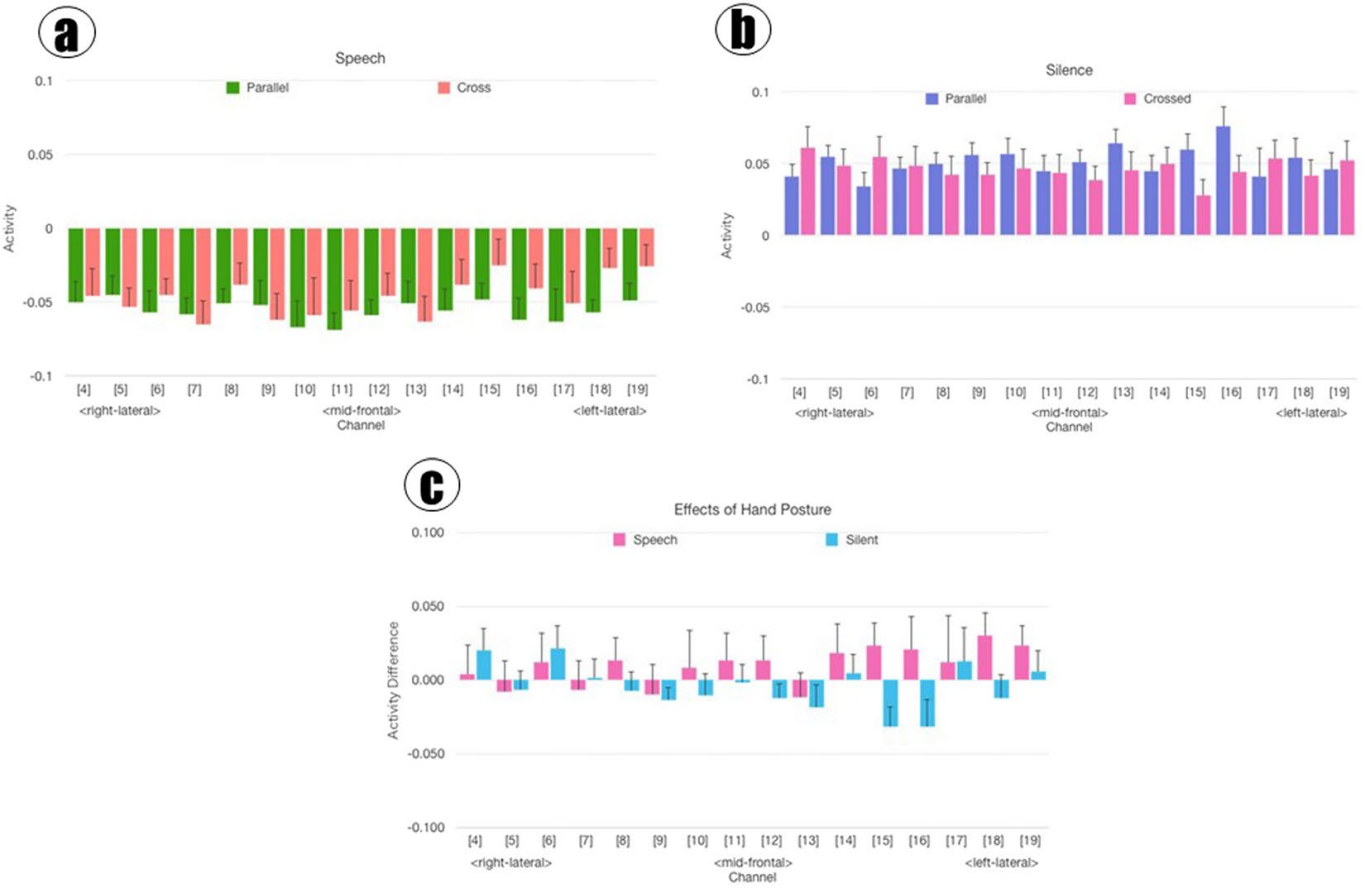
(**a**) Overall prefrontal activities to speech under parallel and crossed hand conditions. The error bars in all figures represent SE (standard error). (**b**) Overall prefrontal activities to silence under parallel and crossed hand conditions. (**c**) Difference between crossed-hand activity and parallel-hand activity for speech and silent stimuli.

Data from individual NIRS channels, averaged across epochs and participants, are shown in Fig. 1. Figure 1(a) shows the data for the speech condition for each of the two hand-posture conditions. Figure 1(b) shows the corresponding results for the silent condition. Figure 1(a) versus 1(b) clearly show that activity during speech is significantly different from that during silence. The effect of hand posture is less clear. In Fig. 1(a), levels seem generally highest for left-lateral channels in crossed posture. Figure 1(c) shows the difference between hand conditions for each channel and for each of the sound conditions.

In the statistical analysis of these data, by a repeated-measures ANOVA with hand posture, stimulus sound condition and brain channels as within-subject factors, the main effect of sound condition (F (1, 16) = 60.528, p < 0.001, *η*^2^ = 0.435) was significant but the effects of hands (F (1, 16) = 0.043, p = 0.838, *η*^2^ < 0.001) and channel (F (15, 240) = 0.631, p = 0.848, *η*^2^ = 0.007) as well as all interactions (F (15, 240) = 0.926, p = 0.535, *η*^2^ = 0.008) were non-significant. Overall, participants showed higher brain activities in silent resting mode than during passive listening to speech (t (16) = 7.778, p < 0.001).

### The effect of hand posture on laterality

**Figure 2:**
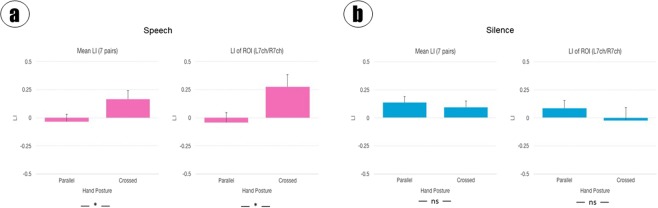
(**a**) LIs to speech. Left: mean LIs of 7 bilateral pair channels. Right: LI of L & R ROIs including 7 channels on each side. (**b**) LIs to silence. Left and Right are as in (**a**).

Laterality of brain activity was examined by calculating laterality indices (LIs) for bilateral channel pairs or for entire bilateral regions of interest (ROIs). The laterality index is the difference between activity on the left and right, normalized by the total activity. Figure 2(a,b) shows two versions of LIs (mean LIs of 7 bilateral channel pairs and LI of L & R ROIs of 7 channels). The 7 pairs of channels in the region of interest excluded the central pair (11 and 12) for the laterality measurements. They revealed that neither posture (F (1, 16) = 1.61/2.06 (mean LI/ROI LI), p = 0.223/0.171, *η*^2^ = 0.015/0.021) nor sound (F (1, 16) = 1.27/0.40, p = 0.277/0.534, *η*^2^ = 0.027/0.014) had a significant main effect on laterality, but they did have a significant interaction (F (1, 16) = 4.97/4.86, p = 0.041/0.043, *η*^2^ = 0.134/0.080). The hand effect was evident only while listening to speech (F (1, 16) = 6.58/6.89, p = 0.015/0.013, *η*^2^ = 0.868/0.873). There was no significant laterality (LI = −0.053/−0.042, 95% CI = [−0.228, 0.123]/[−0.229, 0.145]) with a parallel posture but significant laterality to the left (LI = 0.174/0.277, 95% CI = [−0.036, 0.384]/[0.058, 0.498]) with crossed posture.

The results showed that crossed posture can lead to different results than parallel posture with respect to asymmetric functional connectivity, especially during non-resting state.

## Discussion

As pointed out in the Introduction, it is important for researchers to carefully control study conditions to improve reliability. Previous studies support a connection between arm crossing and various mental functions. Accordingly, the purpose of the present study was to examine the effects of hand/arm posture on the reliability of brain-activity measures associated with listening to speech or silence.

Our results suggested that a stable posture during physiological and psychological experiments is fundamentally important, and that non-stable posture can affect the reliability and reproducibility of the results of physiological testing. Straightforward analysis of results did not reveal any main effects of posture on brain activity as shown by the small effect size in contrast with the significant and large stimulus condition. However, a more sophisticated analysis, dealing with relative function of the left versus right frontal cortex, showed that difference in posture could affect conclusions about laterality of processing associated with listening to speech. The fundamental importance of maintaining stable posture throughout an experiment in order to achieve reliable and replicable results when studying cerebral hemispheric dominance was already recognized by Tsunoda in studies using a key-tapping method (the Tsunoda Test)^4,5^.

Results confirm that hand posture can create a confounding effect on the results of measurements of brain activity and can interfere with the reliability of these measurements. Even when the only task is listening to speech stimuli, participants should be asked to place their hands on a surface and feet on the floor and keep the same stable posture to increase reproducibility of results. Scientists should be aware of this point before experiments, especially on across-hemisphere processing, are first performed and also when verifying their results.

## Conclusion

To achieve accurate reliability and reproductively of results, stable hand posture through the experiment is important.

## Methods

This research protocol (R10-036R) ensured that all experiments were performed in accordance with relevant guidelines and regulations. It was approved by the National Hospital Organization Tokyo Medical Center, Ethics Committee (the institutional review board of Tokyo Medical Center). Before participating in the study, all volunteers provided written informed consent.

### Volunteers

There were 9 Caucasian and 8 Japanese volunteers for this study after those who had previously undergone facial or brain surgery were excluded. The group comprised 9 men and 8 women. This group is consistent with random selection with respect to gender.

### Equipment

Measurements were made with a 22-channel headband-type NIRS device (WOT-220 NIRS, Hitachi, Tokyo, Japan) that was developed to examine oxyhemoglobin levels of the brain, specifically the frontal lobe, via the forehead, since this area is free from obstruction by the hair. The 22 probe channels were attached to the volunteers’ forehead just below the hairline, and the lower-central channel was aligned with Fpz of the International 10–20 system to record and compare activities based on blood oxyhemoglobin levels along the full lengths of the frontal lobes. The participant sat inside a soundproof chamber (AT-81, Rion, Tokyo, Japan). The experimenter was able to monitor the volunteers’ hands and head from outside the chamber through a window. Foot and hands position were monitored with video cameras inside the chamber.

Two computers were used for event-related NIRS recordings. One was used to record NIRS signals with the measurement software of the Hitachi WOT-220 system. The second computer was used for stimulus presentation via Microsoft PowerPoint. A pulse at the start of each stimulus was used to synchronize the data recording with stimulus presentation. This was accomplished using a specially designed device simulating a mouse click in response to the synchronization pulse. Instructions to the volunteers (“parallel” or “crossed” hand position) were recorded at 44,100 samples/sec and presented diotically through EX880ST earphones (Sony Co. Ltd., Tokyo, Japan). The instructions were presented at the same comfortable level, measured as root mean square (rms) value.

### Procedures

To satisfy circasemidian conditions, all volunteers were tested on a weekday afternoon after 2 pm at National Institute of Sensory Organs, National Hospital Organization Tokyo Medical Center. Volunteers sat inside the soundproof booth and the procedures were performed in two successive blocks lasting approximately 18 min in total.

In each of two blocks of data collection, volunteers were asked to maintain the same stable hand posture, either “parallel” or “crossed”. The first block was conducted with “parallel” and the second block of trials with “crossed” hand posture. Exactly the same procedures were performed in both blocks. Data were collected during successive 30-sec epochs in which either auditory speech stimuli or silence was presented. Successive epochs alternated between speech and silence with four repetitions of this sequence. The speech material was a 30-sec segment from an old Japanese fairy tale read by a professional storyteller. None of the volunteers had heard the story before. The sound was presented diotically, i.e. the same sound was presented simultaneously through the earbud earphones to both ears.

To get satisfactorily accurate results and to promote reliability and reproducibility all volunteers were told to obey the following instructions, as in our previous study: (1) sit at the desk and listen to the instruction; (2) keep your hands on the desk and feet on the floor with a stable posture and (3) avoid crossing your legs during the experiment. Furthermore, to confirm that the instructions were followed, each session was monitored via the window from outside the chamber and via the video cameras inside the chamber^8^.

### Analysis

For each epoch, a 30-sec block of 22-channel data was collected, but 6 temporally unstable, channels, namely the most extreme 3 on each side, were excluded. Those channels could not reliably monitor the blood flow in all cases, depending on the forehead width and hair of the volunteers. In the initial stage of processing, the overall average level of activity was calculated for each channel and was used as the baseline for that channel.

We first analyzed the data from individual channels and performed a repeated-measures ANOVA with hand posture, stimulus condition, and brain channels as within-subjects’ factors.

We next calculated laterality indices (LIs) under respective hand conditions and respective stimulus conditions for each of 7 bi-hemispheric pairs of channels, excluding the central pair, which spans the midline. Each LI represents the direction and extent to which activity on one side exceeds the other. The LI is given by

1 $${\rm{LI}}=({\rm{L}}-{\rm{R}})/(|{\rm{L}}|+|{\rm{R}}|),$$

where

L = data from the left (ch13~ch19)

R = data from the right (ch10~ch4)

Two methods were used to calculate the LIs from each participant to indicate the dominance of their verbal hemispheres. In one, LIs were calculated separately for each of the 7 channel pairs and then averaged. In the other, channel activity levels were summed across the 7 channels in the region of interest on each side and the sums were used to calculate the LI.

## Data Availability

The data that support the findings of this study are available from the corresponding author upon reasonable request.
